# Cost saving analysis of prediabetes intervention modalities in comparison with inaction using Markov state transition model—A multiregional case study

**DOI:** 10.1111/1753-0407.13553

**Published:** 2024-04-25

**Authors:** Hussain Abdulrahman Al‐Omar, Marcin Czech, Tran Quang Nam, Ulrike Gottwald‐Hostalek, Nikola Vesic, James Whitehouse, Maddy Dawson

**Affiliations:** ^1^ Department of Clinical Pharmacy College of Pharmacy, King Saud University Riyadh Saudi Arabia; ^2^ Health Technology Assessment Unit (HTAU) College of Pharmacy, King Saud University Riyadh Saudi Arabia; ^3^ Pharmacoeconomic Department Institute of Mother and Child Warsaw Poland; ^4^ Department of Endocrinology University Medical Center Ho Chi Minh City Vietnam; ^5^ Merck KGaA Darmstadt Germany; ^6^ Merck doo Belgrade Serbia; ^7^ Lightning Health Ltd London UK

**Keywords:** budget impact, case study, cost saving, metformin, prediabetes

## Abstract

**Background:**

Prediabetes management is a priority for policymakers globally, to avoid/delay type 2 diabetes (T2D) and reduce severe, costly health consequences. Countries moving from low to middle income are most at risk from the T2D “epidemic” and may find implementing preventative measures challenging; yet prevention has largely been evaluated in developed countries.

**Methods:**

Markov cohort simulations explored costs and benefits of various prediabetes management approaches, expressed as “savings” to the public health care system, for three countries with high prediabetes prevalence and contrasting economic status (Poland, Saudi Arabia, Vietnam). Two scenarios were compared up to 15 y: “inaction” (no prediabetes intervention) and “intervention” with metformin extended release (ER), intensive lifestyle change (ILC), ILC with metformin (ER), or ILC with metformin (ER) “titration.”

**Results:**

T2D was the highest‐cost health state at all time horizons due to resource use, and inaction produced the highest T2D costs, ranging from 9% to 34% of total health care resource costs. All interventions reduced T2D versus inaction, the most effective being ILC + metformin (ER) “titration” (39% reduction at 5 y). Metformin (ER) was the only strategy that produced net saving across the time horizon; however, relative total health care system costs of other interventions vs inaction declined over time up to 15 y. Viet Nam was most sensitive to cost and parameter changes via a one‐way sensitivity analysis.

**Conclusions:**

Metformin (ER) and lifestyle interventions for prediabetes offer promise for reducing T2D incidence. Metformin (ER) could reduce T2D patient numbers and health care costs, given concerns regarding adherence in the context of funding/reimbursement challenges for lifestyle interventions.

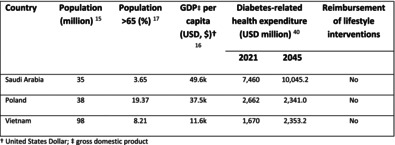

## Graphical abstract

Highlights

- Countries moving from low to middle income are most at risk from the type 2 diabetes (T2D) “epidemic” and may find implementing preventative measures challenging; yet prevention has largely been evaluated in developed countries.
- This study explored costs and benefits of various prediabetes management approaches in Poland, Saudi Arabia, and Vietnam, with the aim of facilitating resource use and planning decisions.
- Pharmacological and lifestyle prediabetes interventions offer promise for reducing T2D incidence. In the context of adherence concerns and funding/reimbursement challenges for lifestyle interventions, metformin alone may be an effective, cost‐saving strategy.

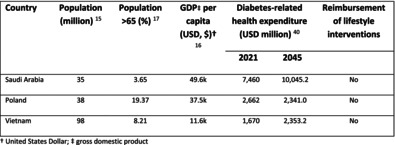

## 1 INTRODUCTION

With ~780 million adults expected to be living with diabetes by 2045, it is a global challenge.^1^ Over 90% of people with diabetes have type 2 diabetes (T2D), characterized by insulin resistance.^2^ T2D leads to various health risks (eg, heart disease, stroke, renal complications, and vision loss), intensifies health care use, and increases medical care costs for patients and governments. The causes of T2D are predominantly lifestyle factors such as poor diet and lack of physical activity. Early intervention and lifestyle modifications can delay or even prevent T2D.^2^

Almost all people progressing to T2D first experience intermediate hyperglycemia or “prediabetes.” Prediabetes is defined as a state of abnormal glucose homeostasis, where blood glucose levels are elevated above those considered normal but not as high as those indicating T2D diagnosis.^3^ Prediabetes can be defined by impaired glucose tolerance (IGT), impaired fasting glucose (IFG) or increased glycated hemoglobin (HbA_1C_); the exact definition varies between organizations. The American Diabetes Association (ADA) defines prediabetes as HbA_1C_ of 5.7%–6.4% and/or impaired fasting blood glucose of 100–125 mg/dL (140–199 mg/dL 2 h after a 75 g oral glucose tolerance test).^2^ The World Health Organization has a narrower definition of fasting blood glucose: 110–125 mg/dL and 2‐h plasma glucose: 140–200 mg/dL.^4^

Prediabetes is associated with increased body weight, increased insulin resistance, and a decline in β‐cell function and may eventually lead to T2D.^3^ Parameters such as age, gender, ethnicity, body mass index (BMI), and a family history of diabetes are risk factors for developing T2D. Although there is no universally accepted T2D prediction model, individuals aged ≥25 y with BMI ≥24 and impaired plasma glucose are considered high risk.^5^

People with prediabetes incur direct medical costs related to glucose testing, increased medical visits, and care associated with early diabetes‐related risk management. A US study found that, annually, people with prediabetes incur 1.8 times the average number of ambulatory visits for endocrine complications and 1.5 times the average number for hypertension.^2^ Indirect costs such as productivity loss, early mortality, absenteeism, and presenteeism also contribute to economic burden.^2^

Globally, prediabetes is considered an important health priority for decision‐ and policymakers for three key reasons. First, prediabetes signifies a high likelihood of progressing to T2D, which imposes significant costs on health care systems. Second, prediabetes is associated with a heightened risk of cardiovascular disease and is a major cause of both mortality and economic burden on health systems. Third, detection of prediabetes, alongside better detection of undiagnosed T2D, can signal interventions to delay or even prevent T2D onset and contribute to overall health and well‐being.^2^

Awareness is growing of the benefits of preventative intervention for prediabetes; pharmacological agents and lifestyle modifications are proven to delay or prevent T2D onset. Metformin is recommended by the ADA for preventing T2D in high‐risk individuals.^3^ The major glucose‐lowering effect of metformin is mediated through inhibition of hepatic gluconeogenesis; however, the exact mechanism of action is still debated.^6^ The first‐line intervention recommended in practice guidelines for prediabetes is intensive lifestyle change (ILC).^3^ With diet changes and increased physical activity, it is possible to achieve normal glucose tolerance (normoglycemia) following prediabetes.^2^ This was demonstrated in a Finnish study,^7^ which found that lifestyle changes (individualized counseling on reducing weight and total/saturated fat intake and increasing fiber intake and physical activity) reduced the risk of diabetes by 58%. However, although lifestyle modifications can reduce T2D risk, high patient adherence is difficult to maintain in the real world. In the United States, for example, 30% of adults do not engage in any regular physical activity.^8^

There is significant interest in developing cost‐effective strategies to prevent prediabetes progression.^9^ A 2018 systematic literature review identified 29 published studies using decision models to predict prediabetes progression and evaluate prevention strategies.^9^ Interventions evaluated included screening, interventions, screening plus intervention, and current care only. The majority of studies (*n* = 24, 83%) reported that interventions were cost effective relative to usual care or no intervention.^9^ This is supported by a further systematic literature review, which identified 23 cost‐effectiveness studies of metformin in prediabetes and found that early intervention in individuals at high risk of developing T2D is cost effective. The study found that ILC is cost effective vs metformin, but metformin may result in cost savings due to the high costs of implementing and maintaining an ILC program. Additionally, for those who cannot practically comply with ILC due to cost constraints or inability to adhere to ILC, metformin was considered an economically feasible option.^5^ Furthermore, a 2017 review of 27 studies in the United Kingdom explored the cost‐effectiveness of lifestyle interventions and metformin in reducing the subsequent incidence of T2D. The study found that metformin and lifestyle programs appear equally cost effective when only the costs of the health system are taken into account, but metformin is more cost effective when costs of participants' time (participating in and traveling to program activities) are included. However, the findings highlighted the heterogeneity of the evidence base in terms of interventions, target populations, and modeling approaches and emphasized the need for further economic evaluation in low‐ and middle‐income countries.^10^ Finally, a research program by the Economist Intelligence Unit investigated potential economic impact of delaying the onset of T2D and found that prediabetes prevention could produce substantial savings, expected to reach between Int$ 16 and Int$ 300 billion (in Brazil, China, Indonesia, Mexico, Russia, and Saudi Arabia) over the next 20 years.^2^ Therefore, there is considerable evidence to suggest that early intervention with pharmacotherapy and/or ILC in individuals at high risk of T2D is cost effective.

Diabetes prevalence has increased in recent decades in most developed and developing countries, and global T2D prevalence is projected to increase to 7079 per 100 000 by 2030.^11^, ^12^ Alongside rising incomes and greater access to processed and unhealthy foods, countries moving from low‐income to middle‐income status will experience the greatest increase in T2D.^13^ Despite this, both direct and indirect costs associated with the prediabetes pathway have largely been evaluated in developed countries.^2^

This study aims to build on existing literature by exploring the cost of prediabetes and benefits of potential intervention strategies across three countries (Poland, Saudi Arabia, and Vietnam; Table 1) with high population prevalence of elevated IGT (6.8%, 12.9%, and 8.9%, of adults aged 20–79 y, respectively in 2021^18^), and contrasting economic status and spending on health care.^14^, ^15^, ^16^, ^19^ The objective was to understand the role of treating prediabetes with established pharmacological/non‐pharmacological modalities and the potential monetary benefits of avoiding more costly disease states (T2D); this may assist health care systems, decision makers and policymakers in the reallocation and prioritization of financial resources.

**TABLE 1:** Comparison of the health care systems in Saudi Arabia, Poland, and Vietnam.

Country	Population (million)^14^	Population > 65 (%)^15^	GDP per capita (USD, $)^16^	Diabetes‐related health expenditure (USD million)^17^		Reimbursement of lifestyle interventions
				2021	2045	
Saudi Arabia	35	3.65	49.6 k	7460	10045.2	No
Poland	38	19.37	37.5 k	2662	2341.0	No
Vietnam	98	8.21	11.6 k	1670	2353.2	No

Abbreviations: GDP, gross domestic product; USD, US dollar.

## 2 METHODS

**FIGURE 1:**
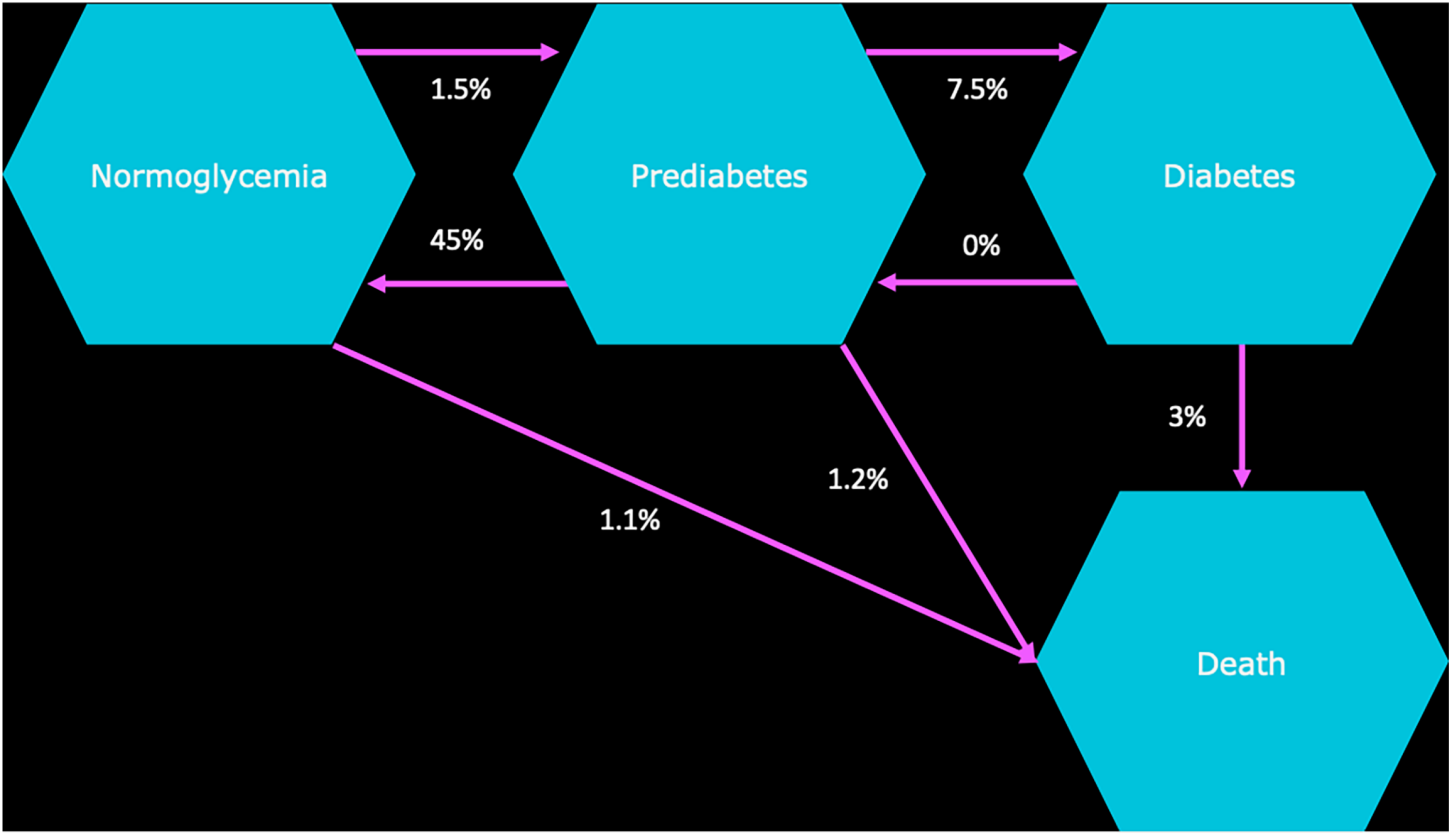
Model structure overview: health state transitions from normoglycemia to death.^2^

A Markov state transition model was developed using an existing framework published in 2020 and validated in consultation with opinion leaders.^2^ The study was conducted using a public health care system perspective for three countries (Poland, Saudi Arabia, and Vietnam), over time horizons of 1, 5, 10, and 15 y. Two main scenarios were compared: “inaction” (with no prediabetes intervention selected) and “intervention.” Intervention scenarios were metformin (extended release [ER]) alone, ILC alone, ILC in combination with metformin (ER), and ILC with metformin (ER) “titration” if noncompliant with ILC. All scenarios were calculated using a cohort Markov state transition model of monthly cycles within the reference framework (Figure 1). Components of ILC were defined via desk research and validated through a separate consultation process between the authorship. Final components were agreed to be the same across all markets for consistency and concluded as health club/gym attendance per month and healthy food budget per month.

The population aged ≥20 y with prediabetes was estimated for each country based on published demographic data, combined with estimates of those with either IFG or IGT (fasting plasma glucose 110–125 or <126 mg/dL; Table 2).^1^, ^22^ The eligible patient population entered the model in the prediabetes disease state, and the cohort was followed for the maximum time horizon of 15 y.

**TABLE 2:** Population and T2D prevalence estimates in Poland, Saudi Arabia, and Vietnam.

Country	Population age range	Total population (inflated from base year 2021)^20^	Annual population growth rate^20^	Prediabetes prevalence estimate	T2D prevalence estimate	Total prevalent population (2022)
Poland	20–29	4 234 204	−0.31%	6.8%^1^	6.8%^1^	287 926
	30–39	5 865 052				398 824
	40–49	5 645 197				383 873
	50–59	4 492 238				305 472
	60+	9 933 086				675 450
	Total	30 169 777				2 051 545
Saudi Arabia	20–29	5 360 331	1.50%	12.8%^1^	18.4%^1^	364 503
	30–39	7 011 184				476 761
	40–49	6 655 298				452 560
	50–59	3 503 340				238 227
	60+	2 210 881				150 340
	Total	24 741 035				1 682 390
Vietnam	20–29	14 994 099	0.85%	13.8%^21^	5.9%^21^	1 019 599
	30–39	16 557 128				1 125 885
	40–49	13 877 325				943 658
	50–59	11 379 085				773 778
	60+	12 646 551				859 965
	Total	69 454 188				4 722 885

Abbreviation: T2D, type 2 diabetes.

### 2.1 Treatment use

In addition to ILC, metformin ER was selected due to its established indication in treating prediabetes in the respective countries.^23^ Adherence to ILC was defined based on annual treatment usage rates for ILC taken from two key published studies and calculated as 49% per year^24^, ^25^ and assuming ILC protocols were the same in each country and considered appropriate based on clinical opinion. It was assumed once a patient defaults on ILC they will either move to inaction (in the ILC alone analysis) or metformin (ER) plus ILC (in the ILC and metformin [ER] titration arm). For combination therapy it was assumed that once a patient defaults on ILC, they remain with the same treatment effect as metformin (ER) alone. Compliance with metformin (ER) was assumed to be 90% throughout the lifecycle of the model,^23^ effective for the first year on treatment. Patients noncompliant with metformin moved to the “inaction” arm.

### 2.2 Clinical input parameters

The annual risk reduction of prediabetes transitioning to T2D among patients using metformin (ER) or ILC were taken from the Diabetes Prevention Program Research Group randomized clinical trial.^26^ The reported 3‐y and 7‐y incidence reduction in both treatment arms was reanalyzed into monthly rates for the purposes of the state transition model using the eigen decomposition method (Table 3).^28^ In combination treatment, the ILC incidence reduction value was carried forward as a conservative estimate of benefit. Other transitions between disease states closely followed the original rates presented in the Economist Intelligence Unit analysis.^2^ This prior analysis assumed no significant differences between countries and therefore carried forward global values when estimating transition rates. It also explored three different scenarios of regression from prediabetes to normoglycemia (15%, 30% and 45%), reflecting the wide range seen in clinical literature (11%–59%), using a default conservative estimate of 45%. The current study used this 45% assumption. Patients surviving in the model beyond 3 y switch transition rates within the model to reflect the outcomes of the Knowler et al study, and adverse intervention effects are excluded.^26^

**TABLE 3:** Clinical parameter inputs: estimated annual transition probabilities.^2^, ^26^

Parameter	Metformin (ER)	ILC
Estimated annual transition of patients from normoglycemia to prediabetes^2^	1.5%	
Estimated annual transition of patients from normoglycemia to T2D^2^	0.72%	
Estimated annual transition of patients from prediabetes to normoglycemia^2^	45%	
Estimated annual transition of patients from normoglycemia to death^2^	1.1%	
Estimated annual transition of patients from prediabetes to T2D^2^	7.5%	
Estimated annual transition of patients from prediabetes to death^27^	1.2%	
Estimated annual transition of patients from T2D to normoglycemia^2^	0%	
Estimated annual transition of patients from T2D to prediabetes^2^	0%	
Estimated annual transition of patients from T2D to death^2^	3%	
Annual T2D risk reduction (<3 years)^26^	31%	58%
Annual T2D risk reduction (>3 years)^26^	18%	34%

Abbreviations: ER, extended release; ILC, intensive lifestyle change; T2D, type 2 diabetes.

### 2.3 Intervention costs/prices

Metformin (ER) average list prices for all available brands were taken directly from published government data sources within each country; cost per mg per month was calculated by applying an average dosing schedule based on prescribing information. ILC components were defined by elements that could carry a cost implication (gym membership and a healthy food budget) and local cost sources were validated through a review process with opinion leaders. For a conservative cost estimate, the maximum dose of metformin ER (2000 mg) was applied across all drug forms. All costs are presented in Table 4 in US dollars (USD) using the spot exchange rate in August 2022.^29^

**TABLE 4:** Intervention costs incurred in Poland, Saudi Arabia, and Vietnam through metformin and intensive lifestyle change.

Intervention	Poland (USD, $)	Saudi Arabia (USD, $)	Vietnam (USD, $)
Metformin (ER) cost per month	5.29	14.65	6.26
ILC cost per month	154.40	273.88	160.74

Abbreviations: ER, extended release; ILC, intensive lifestyle change; USD, US dollar.

## 3 MEDICAL COSTS

**FIGURE 2:**
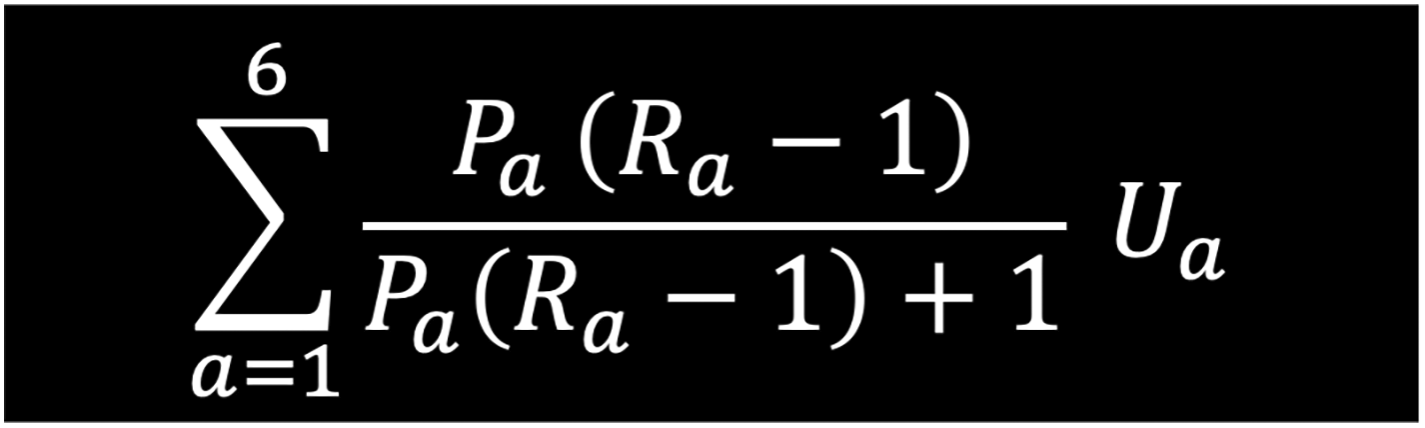
*R* rate. Where, for each age group *a* = {20–29, 30–39, 40–49, 50–59, 60+}, *P*_a_ is the T2D (prediabetes) prevalence rate, *U*_a_ is the total projected health resource use for the given country, and *R*_a_ is the “relative cost ratio” (annual per‐cap expenditures for people with T2D/annual per‐cap expenditures for people without T2D).^3^ T2D, type 2 diabetes.

The costing of disease states within this framework followed closely the attributable fraction cost methodology utilized in the Economist Intelligence Unit analysis.^2^ Specifically, it calculates the ratio of cost inputs between each of the disease states described in Figure 2 (*R* rate). Within the model it is assumed the *R* rate for prediabetes is 1.04 and for T2D is 3.^2^ The equation used is displayed in Figure 2.

Using the World Bank dataset, health care expenditure per capita (US dollars) was estimated and inflated using gross domestic product (GDP) growth rate estimates until 2022.^19^, ^30^ Estimated health care expenditure per capita was applied to each age group. Where it was not possible to find credible data of the estimated health care expenditure between age groups, proxy countries were used to give an accurate representation of resource use as patients progress through life. Total health care expenditure by age group was then adjusted for prediabetes and T2D using the prevalence rates inflated (where necessary) from those defined in Table 5. Total health care expenditure per year within each of the three disease states was than averaged per capita based on the prevalence of the population in each adult age group.

**TABLE 5:** Medical costs incurred in Poland, Saudi Arabia, and Vietnam from patients in different disease states (normoglycemia, prediabetes, type 2 diabetes).^2^, ^19^, ^30^

Disease state	Poland (USD, $)	Saudi Arabia (USD, $)	Vietnam (USD, $)
Normoglycemia	1499	1559	354
Prediabetes (*r* = 1.04)	1559	2056	368
T2D (*r* = 3)	4497	5931	1062

Abbreviations: T2D, type 2 diabetes; USD, US dollar.

### 3.1 Model outputs

The primary model outputs were the difference in patient numbers and cost of the diabetic health state for “inaction” versus “intervention.” Results were displayed for a 1‐, 5‐, 10‐, and 15‐y time horizon, with the total costs across each strategy estimated as the sum of intervention and medical costs.

### 3.2 Scenario analyses

Several one‐way sensitivity analyses were completed to test the robustness of the base case findings across interventions regarding input parameters. This included varying disease state cost +/− 20%, intervention cost +/− 20%, intervention effect +/− 20%, and finally increasing effect of combination treatment by 20% above that of ILC alone. These ranges were selected based on standard plausible ranges that could potentially demonstrate a meaningful difference to base case results.

## 4 RESULTS

### 4.1 T2D patients

**FIGURE 3:**
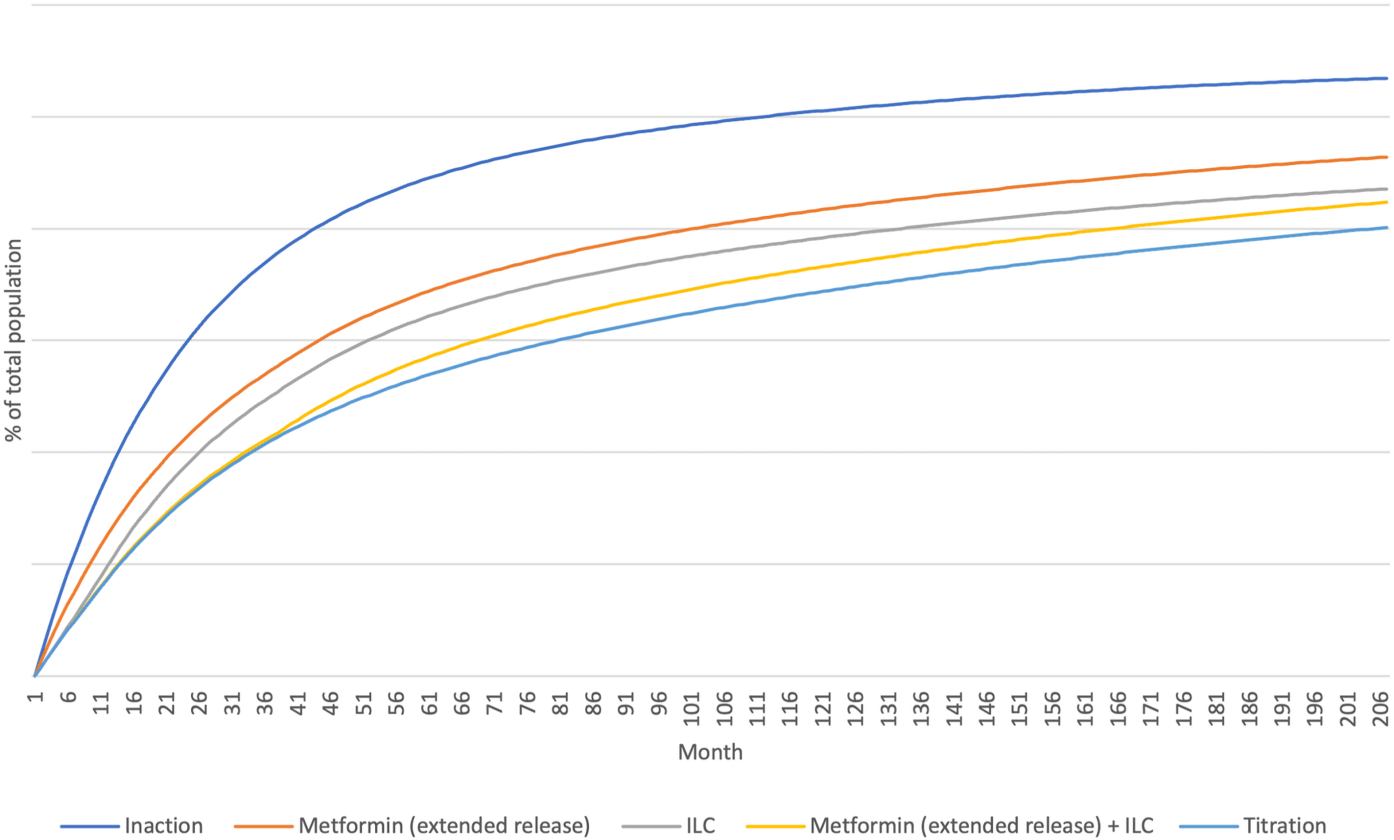
T2D patient distribution with different health states (% of total population in all countries). ILC, intensive lifestyle change; T2D, type 2 diabetes.

Considering state transitions were assumed equivalent across all countries, relative number of patients by state were country agnostic in the analysis. All intervention strategies reduced the number of T2D patients vs no intervention (Figure 3, Table 6), with the most effective ILC + metformin (ER) “titration” (39% reduction at Year 5).

**TABLE 6:** Percentage change in total incident T2D patients at Year 5 by intervention.

Intervention	Percentage change in total incident T2D patients at year 5
Metformin (ER) alone	−21%
ILC alone	−27%
ILC + metformin (ER) combination	−36%
ILC + metformin (ER) titration	−39%

Abbreviations: ER, extended release; ILC, intensive lifestyle change; T2D, type 2 diabetes.

### 4.2 Total health care expenditure

Across the three countries studied, the highest proportion of costs was attributed to the T2D state in all time horizons. Inaction consistently produced the highest proportion of T2D costs, ranging from 9% to 34%.

In all countries the most successful intervention was ILC + metformin (ER) “titration”, in terms of cost reduction in the T2D health care state. However, this did not lead to an overall cost saving for any country, taking into account all disease states and costs. Within the analysis, intervention with metformin (ER) alone was the only treatment strategy that produced a net saving across the time horizon of the model (Tables 8, 9, 10). This was driven by considerably lower intervention cost than ILC, relative effectiveness in preventing T2D vs ILC alone, and the assumption that combination therapy has effectiveness equivalent to ILC alone.

In all three countries and with all intervention strategies vs inaction, cumulative savings increased over time; 15‐y savings with ILC + metformin (ER) “titration” were >$6.4 billion in Poland and Saudi Arabia and $3.5 billion in Vietnam (Table 7). The lower overall cost savings in the T2D state in Vietnam vs other countries were primarily driven by the lower cost of care associated with prediabetes, normoglycemia, and T2D, as well as the generally lower costs of intervention.

**TABLE 7:** Type 2 diabetes cost saving versus inaction in Year 1, 5, 10, and 15 by intervention.

Country	Intervention	T2D cost saving versus inaction (USD, $)			
		Year 1	Year 5	Year 10	Year 15
Poland	Metformin (ER) alone	(93 971 562)	(1 087 775 219)	(2 358 216 477)	(3 467 055 140)
	ILC alone	(155 850 496)	(1 466 726 989)	(3 129 676 880)	(4 648 137 506)
	ILC + metformin (ER) combination	(169 412 084)	(1 857 344 012)	(3 981 098 475)	(5 797 873 181)
	ILC + metformin (ER) titration	(170 761 675)	(1 934 256 951)	(4 347 910 423)	(6 489 236 952)
Saudi Arabia	Metformin (ER) alone	(101 635 894)	(1 176 494 301)	(2 550 552 907)	(3 749 828 590)
	ILC alone	(168 561 682)	(1 586 353 426)	(3 384 933 716)	(5 027 240 181)
	ILC + metformin (ER) combination	(183 229 355)	(2 008 829 221)	(4 305 797 364)	(6 270 748 441)
	ILC + metformin (ER) titration	(184 689 019)	(2 092 015 189)	(4 702 526 539)	(7 018 499 928)
Vietnam	Metformin (ER) alone	(51 088 646)	(591 380 654)	(1 282 069 658)	(1 884 901 679)
	ILC alone	(84 729 792)	(797 401 845)	(1 701 482 372)	(2 527 009 763)
	ILC + metformin (ER) combination	(92 102 695)	(1 009 764 975)	(2 164 366 846)	(3 152 075 883)
	ILC + metformin (ER) titration	(92 836 415)	(1 051 579 518)	(2 363 788 092)	(3 527 943 206)

Abbreviations: ER, extended release; ILC, intensive lifestyle change; T2D, type 2 diabetes; USD, US dollar.

### 4.3 Poland

In Poland, net health care savings vs inaction were seen from Year 5 with metformin (ER) alone. Other interventions were associated with a net cost at all time horizons studied, although the difference declined over time from 65% to 90% more costly than inaction at Year 1 to 3%–8.5% more costly by Year 15. Over 15 y, ILC + metformin (ER) “titration” (the leading strategy in terms of reducing T2D patient numbers) was associated with a health care cost of $4.6 billion (Table 8).

**TABLE 8:** Total health care expenditure versus inaction per intervention within Poland.

	Year 1		Year 5		Year 10		Year 15	
	Total expenditure (USD, $)	% difference versus inaction	Total expenditure (USD, $)	% difference versus inaction	Total expenditure (USD, $)	% difference versus inaction	Total expenditure (USD, $)	% difference versus inaction
Metformin (ER) alone	43 717 967	1.21%	(482 630 941)	−2.64%	(1 252 229 682)	−3.41%	(1 894 533 847)	−3.47%
ILC alone	2 388 641 795	65.85%	3 396 228 581	18.56%	2 680 000 924	7.29%	1 895 684 894	3.48%
ILC + metformin (ER) combination	2 493 223 691	68.73%	3 459 943 730	18.91%	2 586 527 266	7.03%	1 736 663 518	3.18%
ILC + metformin (ER) titration	3 152 167 349	86.9%	6 028 924 949	32.95%	5 439 863 646	14.79%	4 622 835 527	8.47%

Abbreviations: ER, extended release; ILC, intensive lifestyle change; USD, US dollar.

**FIGURE 4:**
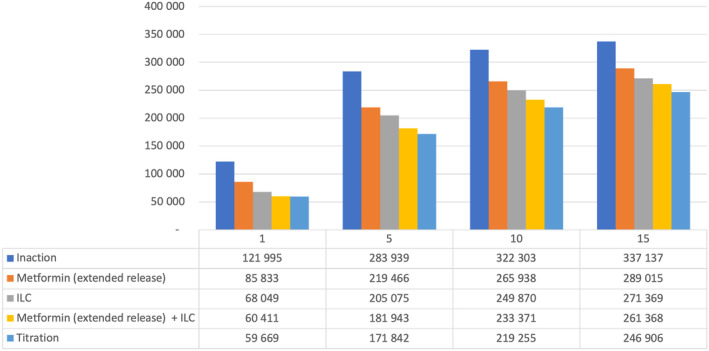
Total estimated patients in the T2D health state by intervention in Poland. ILC, intensive lifestyle change; T2D, type 2 diabetes.

Figure 4 details estimated numbers of T2D patients over time in Poland with the various strategies. In Year 5, ILC + metformin (ER) “titration” reduced the number of T2D patients by >110 000 vs inaction; even the least effective intervention strategy (metformin [ER] alone) led to >60 000 fewer T2D patients vs inaction at Year 5.

### 4.4 Saudi Arabia

In Saudi Arabia, net health care savings vs inaction were seen from Year 5 with metformin (ER) alone. Other interventions were associated with a net cost at all time horizons studied, although the difference declined over time from 90% to 120% more costly than inaction at Year 1 to 6%–14% more costly by Year 15. Over 15 years, ILC + metformin (ER) “titration” (the leading strategy in terms of reducing T2D patient numbers) was associated with a health care cost of $8.3 billion (Table 9).

**TABLE 9:** Total health care expenditure versus inaction per intervention within Saudi Arabia.

	Year 1		Year 5		Year 10		Year 15	
	Total expenditure (USD, $)	% difference versus inaction	Total expenditure (USD, $)	% difference versus inaction	Total expenditure (USD, $)	% difference versus inaction	Total expenditure (USD, $)	% difference versus inaction
Metformin (ER) alone	172 447 662	4.40%	(257 204 309)	−1.3%	(1 060 295 373)	−2.67%	(1 741 093 105)	−2.95%
ILC alone	3 512 611 428	89.53%	5 295 407 823	26.76%	4 642 519 299	11.67%	3 838 161 907	6.5%
ILC + metformin (ER) combination	3 758 801 563	95.81%	5 683 404 693	28.72%	4 933 567 295	12.41%	4 110 575 717	6.96%
ILC + metformin (ER) titration	4 647 804 177	118.47%	9 315 488 854	47.07%	9 037 125 986	22.72%	8 330 928 189	14.11%

Abbreviations: ER, extended release; ILC, intensive lifestyle change; USD, US dollar.

**FIGURE 5:**
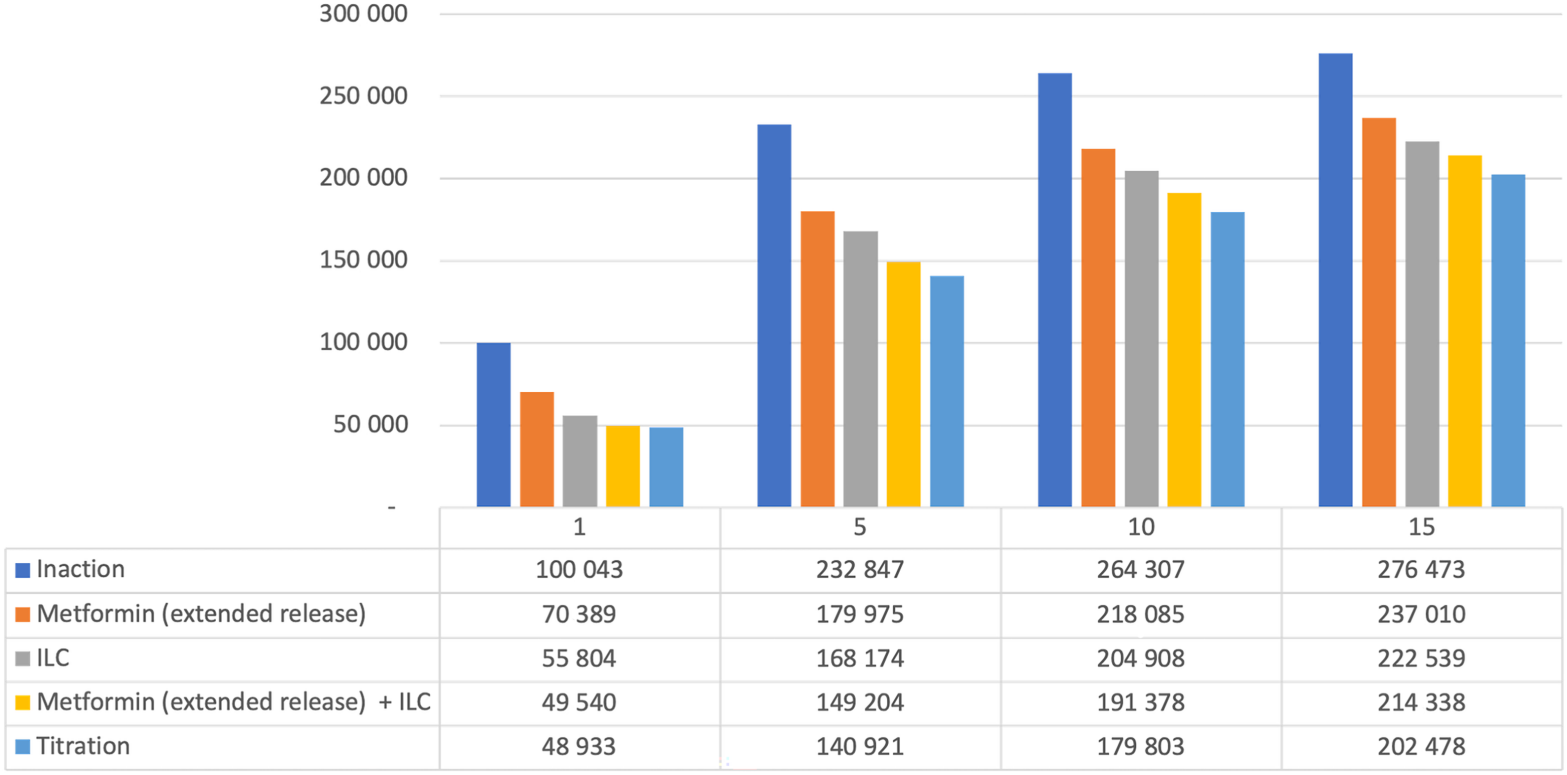
Total estimated patients in the T2D health state by intervention in Saudi Arabia. ILC, intensive lifestyle change; T2D, type 2 diabetes.

Figure 5 details estimated numbers of T2D patients over time in Saudi Arabia with the various strategies. In Year 5, ILC + metformin (ER) “titration” reduced the number of T2D patients by >90 000 vs inaction; even the least effective intervention strategy (metformin [ER] alone) led to >50 000 fewer T2D patients vs inaction at Year 5.

### 4.5 Vietnam

In Vietnam, net health care savings vs inaction were seen with metformin (ER) alone but later than the other countries (Year 10 in this model). Other interventions were associated with a net cost at all time horizons studied, although the difference declined over time from 300% to 400% more costly than inaction at Year 1 to 33%–63% more costly by Year 15. Over 15 years, ILC + metformin (ER) “titration” (the leading strategy in terms of reducing T2D patient numbers) was associated with a health care cost of $18.6 billion (Table 10).

**TABLE 10:** Total health care expenditure versus inaction per intervention within Vietnam.

	Year 1		Year 5		Year 10		Year 15	
	Total expenditure (USD, $)	% difference versus inaction	Total expenditure (USD, $)	% difference versus inaction	Total expenditure (USD, $)	% difference versus inaction	Total expenditure (USD, $)	% difference versus inaction
Metformin (ER) alone	252 919 371	12.82%	222 392 896	02.24%	(142 408 455)	−0.72%	(466 168 841)	−1.57%
ILC alone	5 913 310 120	299.84%	9 903 082 777	99.55%	10 118 401 745	50.61%	9 910 169 302	33.40%
ILC + metformin (ER) combination	6 216 808 865	315.23%	10 605 172 734	106.60%	10 986 415 609	54.96%	10 947 057 993	36.89%
ILC + metformin (ER) titration	7 769 714 719	393.98%	16 805 321 178	168.93%	18 207 356 335	91.08%	18 615 171 962	62.73%

Abbreviations: ER, extended release; ILC, intensive lifestyle change; USD, US dollar.

**FIGURE 6:**
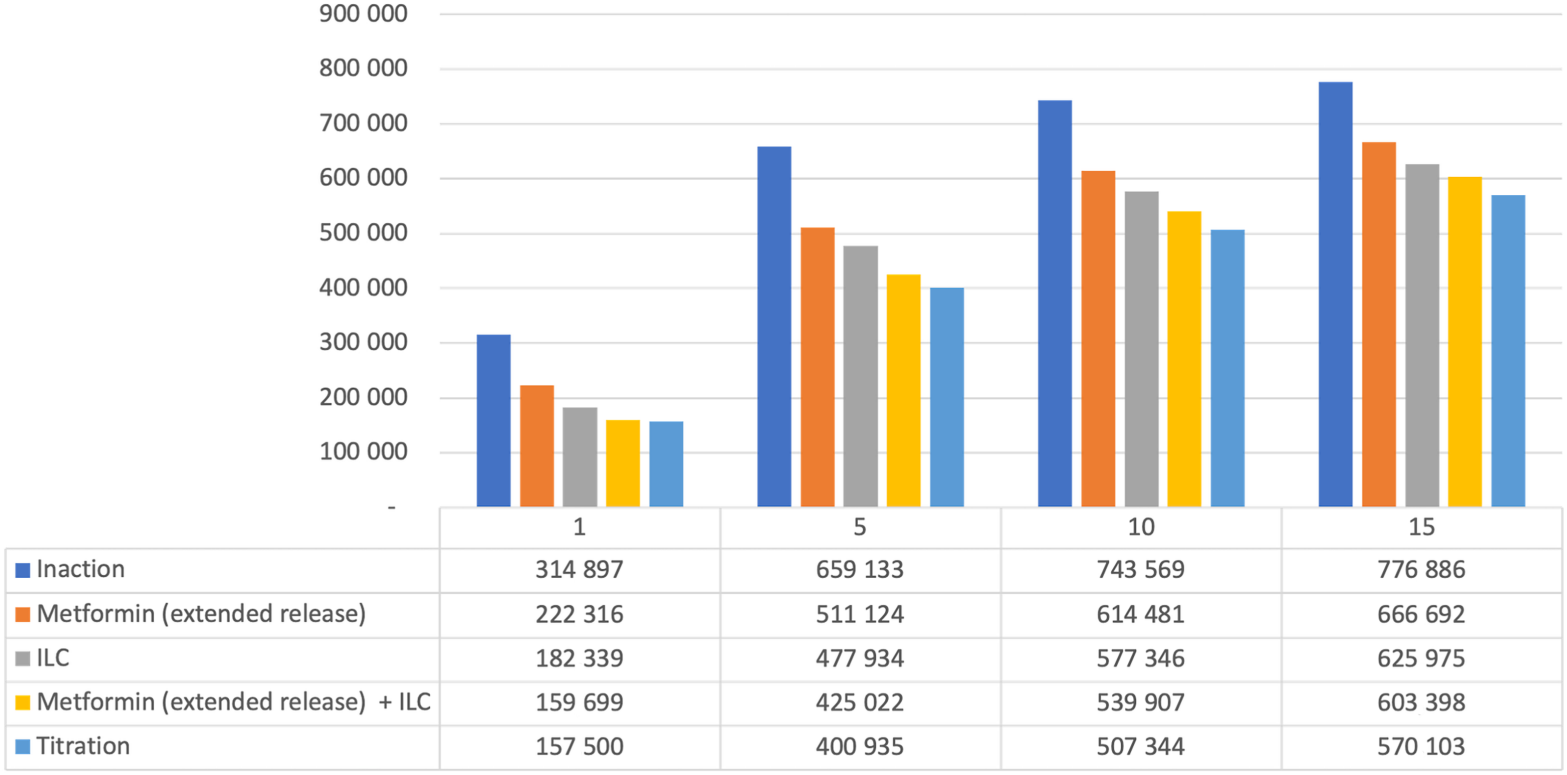
Total estimated patients in the T2D health state by intervention in Vietnam. ILC, intensive lifestyle change; T2D, type 2 diabetes.

Figure 6 details estimated numbers of T2D patients over time in Vietnam with the various strategies. In Year 5, ILC + metformin (ER) “titration” reduced the number of T2D patients by >250 000 versus inaction; even the least effective intervention strategy (metformin [ER] alone) led to >145 000 fewer T2D patients vs inaction at Year 5.

### 4.6 Sensitivity analyses (Year 5 health care expenditure)

**FIGURE 7:**
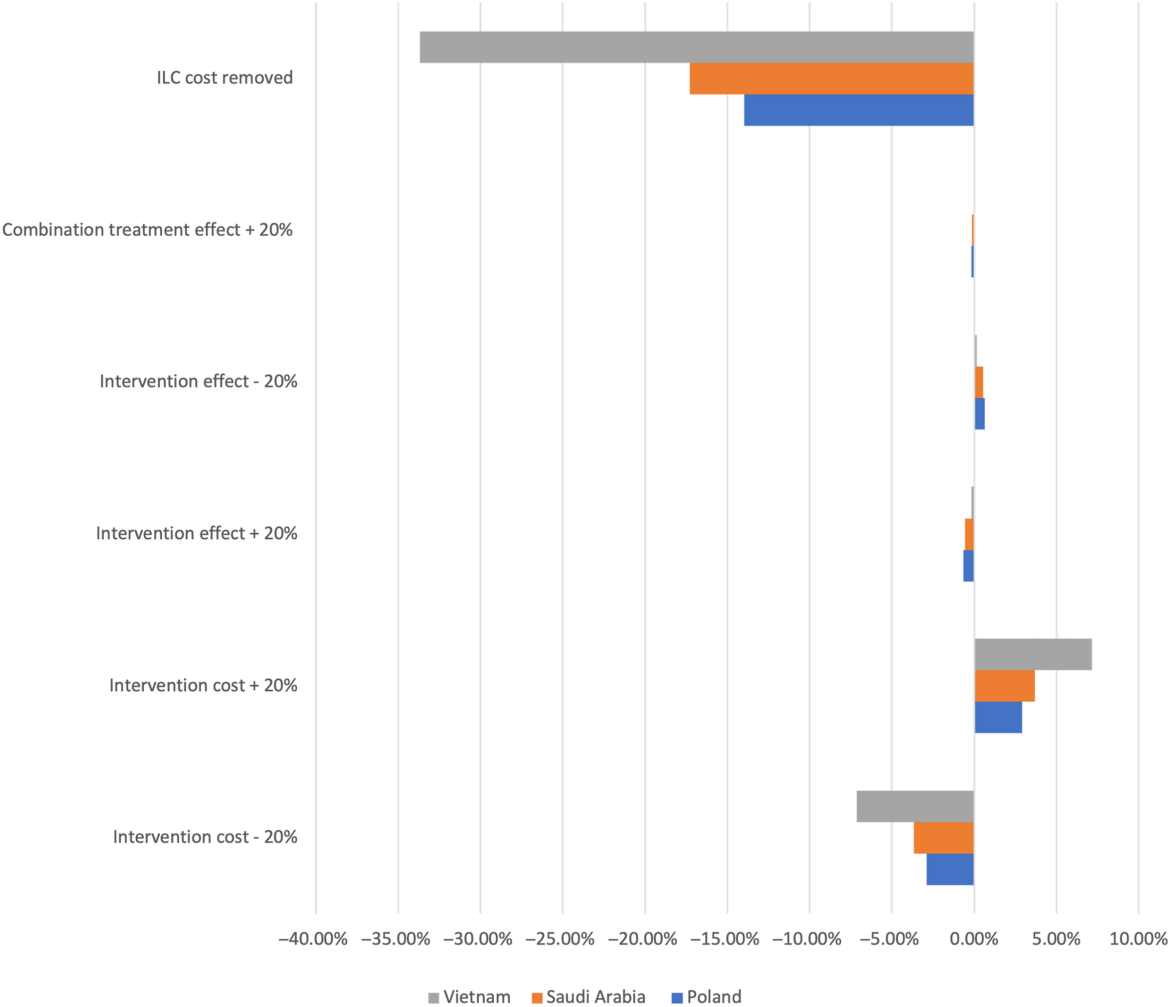
Average effect of one‐way sensitivity analyses on total health care expenditure in Year 5. ILC, intensive lifestyle change.

Of the several one‐way sensitivities that were conducted, removal of ILC cost to the health care system had the largest effect across treatment arms in reducing total health care expenditure vs the base case. The next largest were intervention cost changes, followed by overall 20% adaptations in intervention effects. The largest potential change was observed in Vietnam, where removal of ILC costs to the health care system could potentially lead to an overall expenditure reduction of over 33% compared to the base case in Year 5 (Figure 7).

Vietnam was the country that was most sensitive to changes in intervention cost, with an almost 5% change in total health care expenditure at Year 5 depending on the direction of the scenario. The country that was most sensitive to treatment effect change was Poland, followed by Saudi Arabia. Increasing the effectiveness of combination treatment only had the lowest effect on average across all treatment interventions.

## 5 DISCUSSION

This study suggests the optimal strategy for T2D avoidance is ILC with metformin (ER) “titration” if noncompliant with ILC. However, overall cost remains uncertain, particularly in the early years after adoption. Metformin (ER) alone was the only strategy that produced a net saving to the health care system over the 15‐y time horizon (Tables 8, 9, 10), partly driven by low cost vs other interventions. Sensitivity analyses suggest the cost of ILC has the greatest impact on total health care expenditure, assuming in this case that all costs are retained within the health care system.

Among emerging low‐ and middle‐income countries, access to T2D treatment remains a major cause for concern. Pooled analysis of 55 such countries found that coverage for diet, exercise, and weight loss counseling was 32.2%, 28.2%, and 31.5%, respectively.^31^ Only 4.6% of patients with T2D reported meeting the needs for all treatments recommended to them.^31^

Although ILC has a significant role to play in preventing T2D^32^, ^33^, ^34^ and cost‐effectiveness has been proven, a recent systematic review^10^ raises concern regarding implementation and cost in countries such as the United States. The cost of implementing national prevention programs was 0.13%–0.2% of GDP and net savings were experienced 9–14 years after launch, corresponding with the present analysis.^10^ A key factor is adherence^35^, ^36^, ^37^; key barriers to lifestyle change area) individual evaluation of the importance of initiating lifestyle change; (b) strategies and coping mechanisms for maintaining change; and (c) support in initiating and maintaining change.^35^ Rigorous patient follow‐up has been identified as a solution,^38^ but this could add cost and resource burden. In the absence of adherence, metformin (ER) provides a useful option either in combination or as “titration” treatment^26^, ^38^, ^39^, ^40^ and can be considered cost effective.^10^

The need to invest in prevention presents a paradox for some health care systems, particularly those that need to reduce the burden of T2D management. In some countries such as the United States, there are growing approaches to reimburse lifestyle change, but there remains the question of who pays; behavioral or educational lifestyle management does not always fit into the existing reimbursement model.^3^, ^41^ In the context of the countries studied here, in Vietnam (with a rapidly rising T2D burden^42^, ^43^ and a GDP that is 16% of Saudi Arabia and 21% of Poland),^16^ feasibility of both publicly reimbursed and privately funded ILC is limited, strengthening the need for reimbursed alternatives. Being higher income economies, Saudi Arabia and Poland offer greater potential to fund nonreimbursed alternatives, although the question of adherence remains.

This analysis (as with previous studies) demonstrated the potential of a combined strategy, although across all country interventions only metformin (ER) alone consistently provided a net health care cost saving within the time frame studied. This could change if ILC were reimbursed across countries, so it may be more prudent to discuss overall ILC cost rather than ability to fund specific treatments.

Estimating cost and impact of ILC is challenging; patients will have varying modules within their strategy, influenced by affordability and willingness to adhere. Within this model, ILC cost comprised a healthy eating budget and gym membership. The role of weight loss classes was excluded based on country expert feedback, which may be considered a limitation. Gym membership cost, service standard, and patient acceptability can also vary widely.

Although metformin “titration” as opposed to combination was optimal in this study, it assumes that patients moving from ILC alone to ILC + metformin experience similar effectiveness to combination. Future research should probe the validity of this assumption, as well as effectiveness of combination treatment, which has been assumed similar to ILC alone. Finally, although a step toward country‐specific analysis, country‐specific clinical data on intervention effects could enhance the analysis and expose local differences in metformin (ER) and ILC effectiveness.

## 6 CONCLUSION

Metformin (ER) “titration” if noncompliant with lifestyle intervention alone offers promise in terms of reducing the number of prediabetic adults developing T2D. Although this was not translated to a net health care cost‐saving within a 15‐y model time horizon, it can be expected in the longer term given the well‐documented health and cost benefits of reducing T2D. Metformin (ER) alone offers a potentially useful option to both reduce T2D patient numbers and health care costs, where there are concerns regarding adherence to lifestyle change and in the context of challenges to funding/reimbursement for lifestyle change interventions.

## FUNDING INFORMATION

This research was funded by Merck KGaA, Darmstadt, Germany. Authors Hussain Al‐Omar, Quang Nam, and Marcin Czech did not receive any funding for manuscript development. The authors Quang Nam and Marcin Czech were provided with consultant honoraria for the data generation and analysis only. James Whitehouse and Maddy Dawson declare that Lightning Health was paid for data research and developing the manuscript.

## CONFLICT OF INTEREST STATEMENT

Ulrike Gottwald‐Hostalek and Nikola Vesic are employees of Merck. The views expressed in this article are the authors' own and do not necessarily reflect the views of the company.
